# Biological Activity of *Cupressus sempervirens* Essential Oil

**DOI:** 10.3390/plants12051097

**Published:** 2023-03-01

**Authors:** Lucia Galovičová, Natália Čmiková, Marianna Schwarzová, Milena D. Vukic, Nenad L. Vukovic, Przemysław Łukasz Kowalczewski, Ladislav Bakay, Maciej Ireneusz Kluz, Czeslaw Puchalski, Ana D. Obradovic, Miloš M. Matić, Miroslava Kačániová

**Affiliations:** 1 Institute of Horticulture, Faculty of Horticulture and Landscape Engineering, Slovak University of Agriculture, Tr. A. Hlinku 2, 94976 Nitra, Slovakia; 2 Department of Chemistry, Faculty of Science, University of Kragujevac, 34000 Kragujevac, Serbia; 3 Department of Food Technology of Plant Origin, Poznań University of Life Sciences, 31 Wojska Polskiego St., 60-624 Poznań, Poland; 4 Institute of Landscape Architecture, Faculty of Horticulture and Landscape Engineering, Slovak University of Agriculture, Tr. A. Hlinku 2, 94976 Nitra, Slovakia; 5 Department of Bioenergetics and Food Analysis, Institute of Food Technology and Nutrition, University of Rzeszow, Zelwerowicza 4, 35-601 Rzeszow, Poland; 6 Department of Biology and Ecology, Faculty of Science, University of Kragujevac, 34000 Kragujevac, Serbia

**Keywords:** *Cupressus sempervirens*, MALDI-TOF MS Biotyper, biofilm, insecticidal activity, vapor phase

## Abstract

The aim of this study was to evaluate the antioxidant, antibiofilm, antimicrobial (in situ and in vitro), insecticidal, and antiproliferative activity of *Cupressus sempervirens* essential oil (CSEO) obtained from the plant leaf. The identification of the constituents contained in CSEO was also intended by using GC and GC/MS analysis. The chemical composition revealed that this sample was dominated by monoterpene hydrocarbons α-pinene, and δ-3-carene. Free radical scavenging ability, performed by using DPPH and ABTS assays, was evaluated as strong. Higher antibacterial efficacy was demonstrated for the agar diffusion method compared to the disk diffusion method. The antifungal activity of CSEO was moderate. When the minimum inhibitory concentrations of filamentous microscopic fungi were determined, we observed the efficacy depending on the concentration used, except for *B. cinerea* where the efficacy of lower concentration was more pronounced. The vapor phase effect was more pronounced at lower concentrations in most cases. Antibiofilm effect against *Salmonella enterica* was demonstrated. The relatively strong insecticidal activity was demonstrated with an LC50 value of 21.07% and an LC90 value of 78.21%, making CSEO potentially adequate in the control of agricultural insect pests. Results of cell viability testing showed no effects on the normal MRC-5 cell line, and antiproliferative effects towards MDA-MB-231, HCT-116, JEG-3, and K562 cells, whereas K562 cells were the most sensitive. Based on our results, CSEO could be a suitable alternative against different types of microorganisms as well as suitable for the control of biofilms. Due to its insecticidal properties, it could be used in the control of agricultural insect pests.

## 1. Introduction

Essential oils (EOs) constitute only a fraction of the total plant mass, but they are responsible for significant properties of aromatic plants. Composed of hundreds of biologically active compounds, EOs are effective due to their complex composition. Hydrocarbons such as sesquiterpenes and monoterpenes are dominant, while among oxygenated compounds, aldehydes, ketones, phenols, oxides, alcohols, and acetates are the most abundant. Esters and ethers can also be found in high amounts. Hydrocarbons and oxygenated compounds significantly influence the odor and taste characteristics of EOs [1]. EOs are considered safe substances and can be used as potential antimicrobials [2]. It has also recently been found that EOs can show comparable, and in some cases even higher, efficacy than currently used antimicrobials [3,4].

*Cupressus sempervirens* L. [Cupressaceae] is an evergreen tree with a distinctive aroma, rich in essential oils. It has been used for centuries in traditional medicine for its expectorant, antiseptic properties. It is most commonly used to treat coughs, bronchitis, diabetes, boils, and laryngitis, but also inflammation and toothache [5]. *C. sempervirens* also exhibit significant biological activities including antioxidant, antimicrobial, and insecticidal effects [6].

Today, food safety is emphasized by manufacturers, regulators, and consumers alike [7]. Despite significant modernization of production techniques, contamination of food with food spoilage-causing pathogens and microorganisms is a global problem [8]. Microbial burden poses a risk to food sustainability as well as alimentary diseases, increasing the burden on public health [9]. In this regard, EOs can be a useful alternative for applications in food preservation. 

A biofilm is an organized cluster of microorganisms surrounded by an extracellular polymeric matrix that they produce. The problem with biofilms is that they reduce the effectiveness of antibiotic therapy [10]. Biofilm formation is a mechanism of survival in adverse conditions, and for this reason, it is almost ubiquitous [11]. Literature data show that biofilm formation is influenced, among others, by the characteristics of the contact surface [12]. Biofilms can cause many problems in different industries, including food, medicine, and agriculture.

There are numerous conventional techniques for identifying bacteria, and many of them can be time-consuming, complicated, and expensive [13,14,15]. Considering the urgency of antibiotic resistance and the struggle with bacterial biofilms, the development of quick and accurate techniques is of high importance [15,16]. In recent years, the MALDI-TOF MS technique has been widely used in microbiology to study different types of bacteria (Gram-positive, Gram-negative, mycobacteria, and anaerobic). The advantages of this method are mainly reflected in its precision, speed, simplicity, and reproducibility, as well as in its price [13,14,15]. 

This study aimed to evaluate the antioxidant, antibiofilm, antimicrobial, insecticidal, and antiproliferative activity of *Cupressus sempervirens* essential oil (CSEO). Preliminary testing regarding the antimicrobial activity of the CSEO was performed with disc diffusion and minimum inhibitory concentration assays. To further elucidate the antimicrobial effects of this EO, we have performed an *in situ* assay on kohlrabi as a food model. This assay is performed to assess its potential application as a natural preservative. To our knowledge, there are no previous reports on this species of EO that demonstrate its antimicrobial efficiency in food preservation with an *in situ* assay. Next, we examined the antibiofilm activity of CSEO against *Salmonella enterica* biofilms formed on stainless steel and plastic surfaces using MALDI-TOF MS Biotyper. Additionally, since literature data was lacking, using the MTT assay we determined the antiproliferative activity of this EO towards human breast cancer (MBA-MB-231), human colon cancer (HCT-116), human choriocarcinoma (JEG-3), and chronic myelogenous leukemia (K562). For purpose of examining the biocompatibility of the tested EO, we also evaluated the effects of CSEO on the healthy human lung fibroblast (MRC-5) using the same assay. Finally, by employing GC and GC/MS techniques we have determined the chemical composition of the constituents contained in CSEO. 

## 2. Results

### 2.1. Chemical Composition

Results presented in Table 1 show the volatile composition of *C. sempervirens* essential oil (CSEO). Thirty-nine compounds were identified, which represents 98.9% of the total. Monoterpene hydrocarbons were present in high abundance (90.7%), with α-pinene (40.5%), and δ-3-carene (24.4%) as the major compounds. Other identified monoterpene hydrocarbons were α-terpinolene (8.6%), limonene (4.3%), β-myrcene (3.0%), sabinene (2.1%), and β-pinene (1.7%). From the class of oxygenated monoterpenes (2.4% of the total), monoterpene alcohol, 4-terpineol, was detected with an abundance of 1.9% of the total. Moreover, from the class of sesquiterpenes (5% of the total), sesquiterpene alcohol, cedrol, was dominant (2.4%). Other compounds present in this EO sample were detected in quantities below 1.5%.

### 2.2. Antioxidant Activity

The antioxidant potential of CSEO was determined by the means of the neutralization of stable DPPH radical and ABTS radical cation. The obtained results are presented in Table 2. Based on the IC_50_ value, the radical scavenging capacity of the tested EO was found to be stronger than that of the reference compound Trolox. 10 μL of this EO was able to neutralize 76.32 ± 0.43% of DPPH radical, which is equivalent to the 6.46 ± 0.18 TEAC, and 91.53 ± 0.16% of ABTS radical cation (4.92 ± 0.06 TEAC). 

### 2.3. Antimicrobial Activity In Vitro

#### 2.3.1. Disc Diffusion Method

A weak antimicrobial activity was observed towards one gram-negative bacterium–*P. aeruginosa* (zone of inhibition of 4.33 ± 1.15 mm), and two gram-positive bacteria–*S. aureus* and *E. faecalis* (inhibition zones of 2.67 ± 0.58 mm and 1.00 ± 0.00 mm, respectively). A weak antimicrobial activity was also observed against the yeast *C. krusei* with a zone of inhibition of 3.67 ± 0.58 mm and the biofilm-producing bacterium *S. enterica* with a zone of inhibition of 4.00 ± 1.00 mm. Moderate antimicrobial activities were detected against two gram-negative bacteria, *Y. enterocolitica* (5.67 ± 0.58 mm) and *S. enterica* (6.67 ± 0.58 mm), two species of yeast, *C. glabrata* (8.33 ± 0.58 mm) and C. *tropicalis* (6.33 ± 0.58 mm), and against all the microscopic filamentous fungi tested, the zones of inhibition ranged from 6.33 ± 0.58 mm for *A. flavus* to 7.67 ± 0.58 mm for *P. citrinum*. A strong antimicrobial effect was observed for the gram-positive bacterium *B. subtilis* (10.33 ± 1.53 mm) and the yeast *C. albicans* (11.33 ± 1.15 mm). Further details on the antimicrobial activity are summarized in Table 3.

#### 2.3.2. Minimal Inhibition Concentration

Low MIC 50 (40.92–93.80 µL/mL) and MIC 90 (82.87–131.49 µL/mL) values were detected for two gram-negative bacteria, *Y. enterocolitica* and *S. enterica*, and two yeasts, *C. krusei,* and *C. tropicalis*. Intermediate MIC 50 (154.31–187.31 µL/mL) and MIC 90 (199.21–289.10 µL/mL) values were observed for *C. glabrata* and the biofilm-producing bacterium *S. enterica*. High MIC 50 (202.11–374.02 µL/mL) and MIC 90 (397.64–401.67 µL/mL) values were observed for *P. aeruginosa*, *B. subtilis*, *S. aureus*, *E. faecalis* and *C. albicans*. Compared to the results obtained with the disk diffusion method, a higher efficiency against gram-positive bacteria and biofilm-producing bacteria was observed in the agar microdilution method. Further details of the minimum inhibitory concentrations are summarized in Table 4.

The minimum inhibitory concentrations of CSEO against three species of microscopic filamentous fungi (*Penicillium citrinum*, *Botrytis cinerea*, and *Aspergillus flavus*) were evaluated by a different method because mycelial growth is difficult to observe in the agar microdilution method. A modification of the disk diffusion method was used where the inhibition zones formed around the disk were measured when different concentrations of CSEO diluted in DMSO were applied. The largest inhibition zone was recorded against *P. citrinum* at the highest concentration tested, 500 µL/mL (7.67 ± 1.53 mm), and with decreasing CSEO concentration the inhibition zones decreased resulting in the inhibition zone of 6.33 ± 1.53 mm at a concentration of 62.5 µL/mL. A more pronounced effect was observed against *B. cinerea* when lower concentrations were used. The largest inhibition zone of 8.67 ± 1.15 mm was observed at an applied concentration of 62.5 µL/mL, and the highest concentration tested, 500 µL/mL, an inhibition zone of only 4.67 ± 0.58 mm was observed. Against *A. flavus*, the highest inhibition zone of 7.33 ± 0.58 mm was observed at the highest CSEO concentration of 500 µL/mL, and the inhibition zones decreased as the concentration decreased. A more detailed summary of the results is shown in Table 5.

### 2.4. In Situ Antimicrobial Activity

In situ antifungal activity analysis demonstrated the efficacy of all tested concentrations against microscopic filamentous fungi, while the efficacy of lower tested concentrations was higher. In situ antibacterial efficacy was demonstrated for all bacterial and yeast species except the biofilm-producing bacterium *S. enterica,* for which only low efficacy was observed at the lowest concentration of 62.5 μL/mL, and growth stimulation occurred at the other concentrations tested. For most of the bacteria and yeasts, as with the microscopic filamentous fungi, we observed higher efficacy with the application of the lower concentration of CSEO. More detailed results are shown in Table 6.

### 2.5. Antibiofilm Activity

The MALDI-TOF MS Biotyper mass spectrometer was used to analyze the differences in mass spectra of the biofilm-producing bacterium *S. enterica*. The anti-biofilm effect of CSEO was evaluated on days 3, 5, 7, 9, 12, and 14 in biofilms developing on stainless steel and plastic surfaces and compared with the control biofilms developing without EO treatment. The control planktonic spectra were used for comparison with the experimental groups from the different surfaces since the evolution of the planktonic control spectra was identical to the control spectra on the surfaces analyzed on each day.

**Figure 1:**
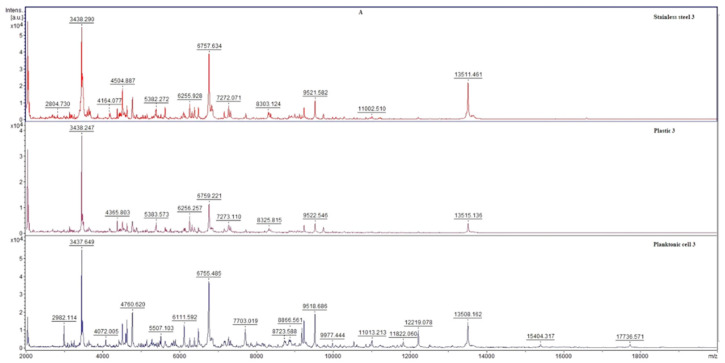

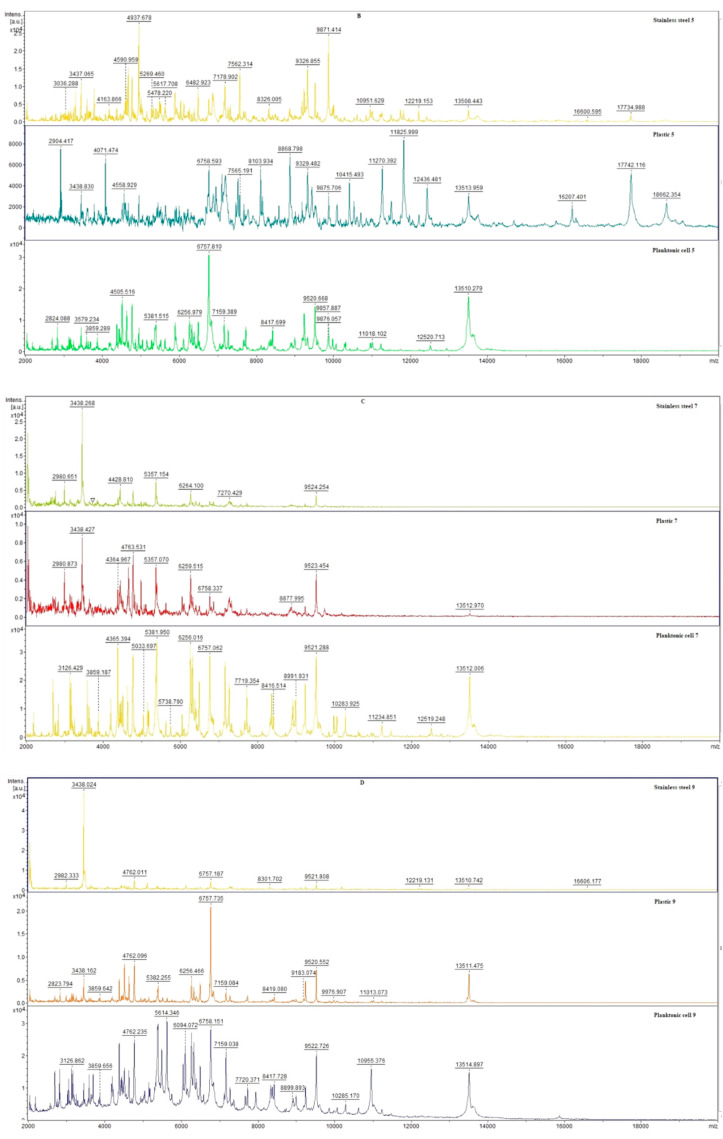

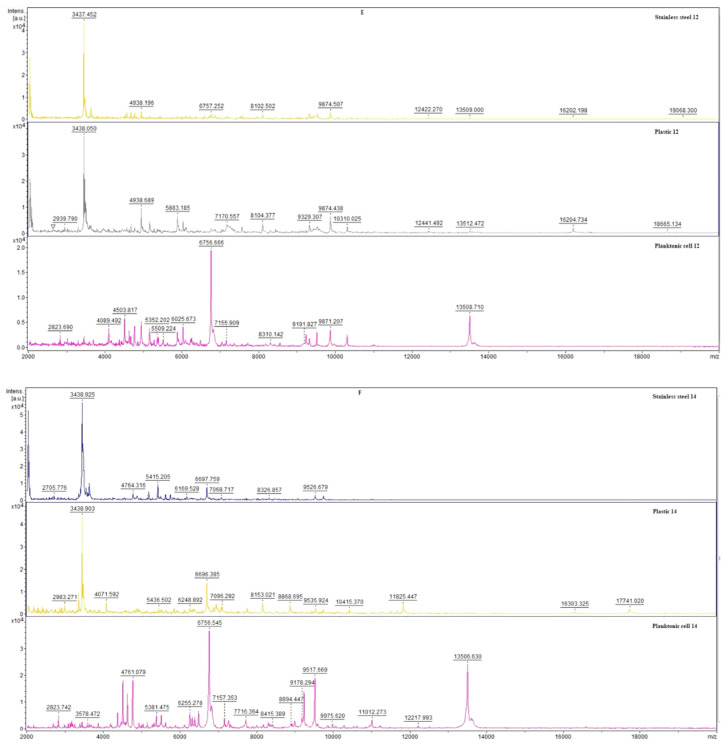
Representative MALDI-TOF mass spectra of *S. enterica*: (**A**) 3rd day, (**B**) 5th day, (**C**) 7th day, (**D**) 9th day, (**E**) 12th day, and (**F**) 14th day.

The development of the primary biofilm of the experimental group during day 3 (Figure 1A) was almost identical to that of the control group; no significant changes were observed in the mass spectra of the two groups. As the development progressed to day 5 of the experiment (Figure 1B), there was a difference between the experimental and control groups suggesting an influence of CSEO on *S. enterica* biofilm homeostasis. From day 7 of the experiment to the end of the experiment (Figure 1C–F), the changes in the experimental groups were significant. Significant visual differences can be observed between the experimental and control spectra. Based on these findings, we conclude that CSEO has a significant disrupting effect on the homeostasis of *S. enterica* biofilm thereby leading to its inhibition. Since the biofilm stage (day 5) was already relatively early affected, CSEO has a significant potential to be applied as an anti-biofilm agent.

**Figure 2:**
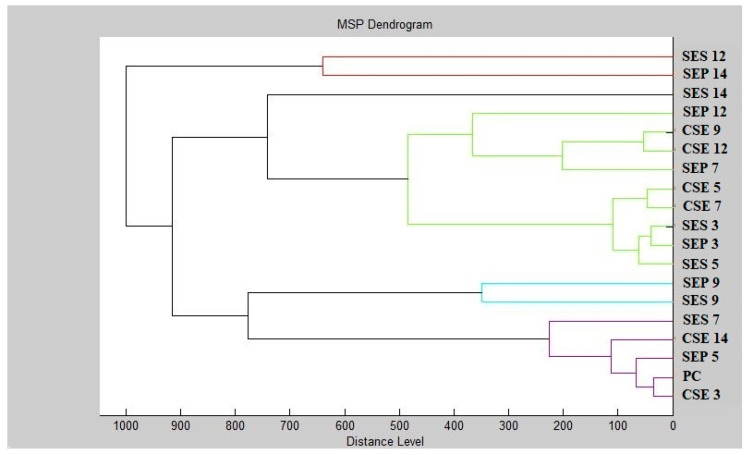
Dendrogram of *S. enterica* biofilm progress after CSEO exposition. SE–*S. enterica*; C-control; S–stainless-steel; P-plastic; PC-planktonic cells.

The dendrogram constructed from the individual MSP distances of the control and experimental mass spectra serves as a visualization of the interrelatedness of biofilms on different surfaces (Figure 2). The constructed dendrogram shows that the shortest MSP distances between the control and experimental groups were observed during days 3 and 5 of the experiment (SES 3;5 and SEP 3;5) along with the control groups throughout the length of the experiment indicating that very little change in the molecular structure of the biofilm was occurring at this time under the influence of CSEO. From day 7 of the experiment (SES 7 and SEP 7) onwards, there was a significant lengthening of the MSP distances of the experimental group from the controls, which is evidence of the inhibitory effect of CSEO against the *S. enterica* biofilm. This trend persisted for the rest of the duration of the experiment. The most significant increases in MSP distances for the experimental groups were observed during days 12 and 14 of the experiment (SES 12;14 and SEP 12;14) indicating significant biofilm inhibition. Based on this evidence, we conclude that CSEO has the potential to act as an antibiofilm agent against *S. enterica* biofilm which confirms the findings inferred from the mass spectra analysis.

### 2.6. Insecticidal Activity of CSEO

CSEO showed relatively strong insecticidal activity (Table 7) towards *O. lavaterae* with the highest concentration tested showing 100% insecticidal activity, killing all individuals, a concentration of 50% (prepared by dilution in 0.1% polysorbate solution) had 80% insecticidal activity and even a concentration of 25% CSEO had more than 50% insecticidal activity killing half of the individuals. At the lower concentrations tested, insecticidal activities of 36.66% and 10% were observed. The lowest concentration tested 3.125% did not show insecticidal activity as well as the control group in which all individuals survived. Out of the obtained results we have calculated lethal concentrations that are found to be for LC50 21.07% and for LC90 78.21%. 

### 2.7. Cell Viability Assay

**Figure 3:**
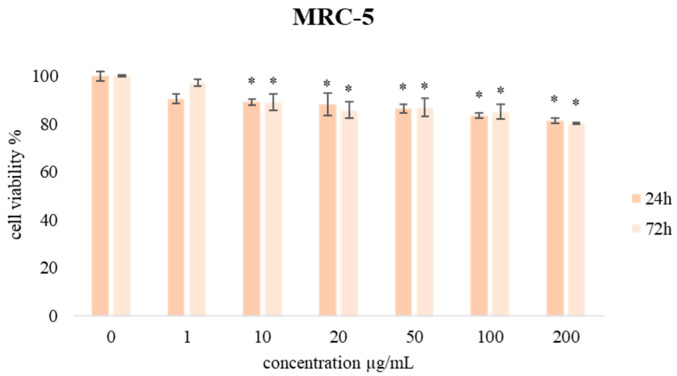
The effects of six concentrations of CSEO on MRC-5 cell viability after 24 h and 72 h of treatment. Results are presented as the mean of three independent experiments ± standard error; * *p* < 0.05 relative to control.

**Figure 4:**
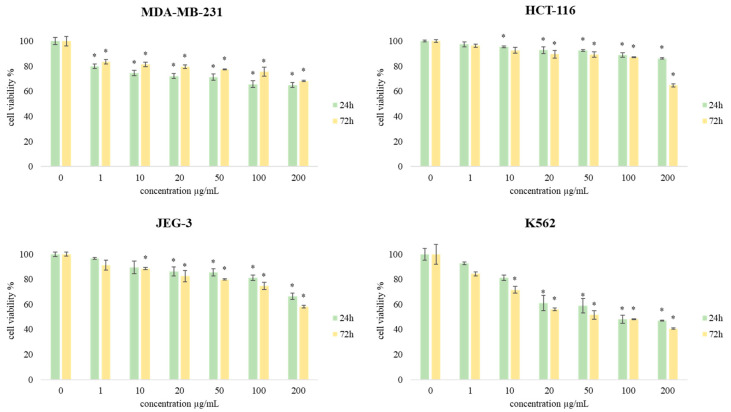
The effects of six concentrations of CSEO on MDA-MB-231, HCT-116, JEG-3, and K562 cell viability after 24 h and 72 h of treatment. Results are presented as the mean of three independent experiments ± standard error: * *p* < 0.05 relative to control.

Additionally, the MTT assay was used to evaluate the effects of CSEO on the viability of human breast cancer (MBA-MB-231), human colon cancer (HCT-116), human choriocarcinoma (JEG-3), chronic myelogenous leukemia (K562), and normal human lung fibroblast (MRC-5) cell lines. During the treatment that lasted 24 h and 72 h, the cells were treated with different concentrations of CSEO (1, 10, 20, 50, 100, and 200 μg/mL), and obtained results are presented in Figure 3 and Figure 4.

The selectivity of the CSEO toward cancer cells was examined by treatment of normal human lung fibroblast cell line MRC-5. Obtained results are presented in Figure 3. The proliferation level of cultivated MRC-5 cells was higher than 80.25% compared to non-treated cells in a concentration of 200 µg/mL, demonstrating that the biocompatibility of CSEO is acceptable. Accordingly, this analysis demonstrated that the tested essential oil did not have a non-specific toxic effect. These results qualify this EO sample as suitable for further evaluation of its antiproliferative effects.

Figure 4 shows the results of cell viability testing of the human breast cancer (MBA-MB-231), human colon cancer (HCT-116), human choriocarcinoma (JEG-3), and chronic myelogenous leukemia (K562) cell lines. Overall, results indicate that all treatments exert time and dose-dependent decrease in cell viability in all cell lines, but the most sensitive was Kthe 562 cell line (47.07%). Considering the presented results, a general conclusion can be made that CSEO induces a significant antiproliferative effect in the tested cells. 

Comparing the results, it can be concluded that K562 cells were the most sensitive to the treatment with CSEO, suggesting that the tested essential oil could be most effectively used for the treatment of chronic myelogenous leukemia. Towards K562 cells, IC_50_ values below the maximal tested concentration of 200 µg/mL were observed for both treatment times-24 h (105.06 µg/mL) and 72 h (66.56 µg/mL). 

For all six tested concentrations of CSEO, the viability of exposed cancer cells was significantly decreased compared to the control cells, and multifold lower when compared to non-cancer human lung fibroblast cells MRC-5. In addition, the cell viability after 72 h was further decreased when compared to short-term treatment, suggesting a time-dependent effect. 

## 3. Discussion

A large number of scientific findings dealing with the chemical composition of EO concluded that the amount of volatiles present in plant matter varies depending on the region in which the plant was growing, the extraction method used, the genetic background of the species, as well as environmental factors such as altitude, climate, soils, and precipitations [17]. Previous studies on *C. sempervirens* essential oil chemical composition reported 10–67 compounds identified depending on the plant organ used for EO extraction [17,18,19,20,21,22,23,24,25,26,27,28,29,30,31]. EOs obtained from the leaves of this species are characterized by α-pinene and δ-3-carene as major components [17,18,21,22,24,28,31]. These results are in agreement with the ones obtained in this study. Considering the volatiles present in minor amounts, our sample was characterized by a notable amount of α-terpinolene, limonene, β-myrcene, sabinene, and cedrol. Selim et al. reported limonene and α-terpinolene being the next most abundant compounds, while some other studies show a high abundance of cedrol, α-terpenylacetate, myrcene, and β-caryophyllene [17,21,22,24]. 

The tests commonly used to examine the antioxidant capacity of essential oils are DPPH and ABTS radical scavenging assays. The presence of compounds with different functional groups in complex mixtures such as EOs as well as the analytical method used can lead to varying results. Previous reports indicate moderate to the strong antioxidant potential of the *C. sempervirens* EOs. Ben Nouri et al. determined the strong potency of this EO toward both DPPH radical and ABTS radical cation [18]. However, some other authors showed moderate radical-scavenging activity toward the DPPH radical [29,32,33,34]. The results obtained in this study show that 10 μL of CSEO can neutralize 76.32 ± 0.43% of DPPH radical, and 91.53 ± 0.16% of ABTS radical cation (4.92 ± 0.06 TEAC). Compared to the IC_50_ value of the standard Trolox which is estimated at 4.39 ± 0.13 mL/L for DPPH radical and 2.96 ± 0.01 mL/L for ABTS radical cation we can conclude that the sample investigated in this study is a strong antioxidant against DPPH radical and ABTS radical cation. The antioxidant activity of essential oils is mainly described as a synergistic or antagonistic effect of two or more of its components [18].

Mazari et al. [22] determined the diameters of inhibition zones including the paper disc (6 mm) for *P. aeruginosa* (7 mm), *E. faecalis* (9 mm), *S. aureus* (10.3 mm), *B. cereus* (7.6 mm), and *E. coli* (9.3 mm) after application of CSEO. Considering that the authors measured the diameter of the inhibition zone using a disc (6 mm) and in our study, the radius of the inhibition zone was measured from the edge of the disc to the edge of the zone so the inhibition zones detected by us for *P. aeruginosa* are larger than those in the referenced study. We detected similar inhibition zones for *E. faecalis* and *S. aureus.* Elansary et al. [29] also evaluated the antimicrobial activity of CSEO by disc diffusion method by measuring the diameter of inhibition zones with the disc (5 mm) and detected inhibition zones for *P. aeruginosa* no inhibition zone was observed, *B. subtilis* (18 mm), *S. aureus* (13 mm). Again, if we consider that the authors measured the diameter, and in our work, the radius was measured, the zones of inhibition reported in the mentioned paper were larger compared to our study except for *P. aeruginosa* in the case of which we determined an inhibition zone of 4.33 mm. Layal et al. [35] determined the diameters of the zones of inhibition in the disc rim (5 mm) for *P. aeruginosa* (7 mm) and *S. aureus* (12 mm) after the application of CSEO. The inhibition zones detected by us were higher for *P. aeruginosa* and lower for *S. aureus*. The differences between our findings and the results of other authors may be due to the different origins of the microorganisms as well as the different origins of CSEO and its acquisition method. 

Emami et al. [31] analyzed the antifungal activity of CSEO on 8 microscopic filamentous fungi (*Fusarium culmorum*, *Fusarium oxysporum*, *Fusarium equisiti*, *Fusarium verticillioides*, *Fusarium nygamai*, *Botrytis cinerea*, and *Microvar. Alternochium* var. *nivale*), and against all the tested species strong antifungal effects were observed at low concentrations. These findings are consistent with our results which reflect a significant antifungal activity of CSEO against microscopic filamentous fungi. Tekaya-Karoui et al. [36] analyzed the antifungal effect of CSEO against 10 species of microscopic filamentous and also reported strong antifungal effects. In agreement with our findings, the authors concluded that CSEO has a significant potential to act as an antifungal agent. Mazari et al. [22] determined the antifungal activity of CSEO against 3 species of filamentous microscopic fungi (*Aspergillus flavus*, *Fusarium oxysporum*, and *Rhizopus stolonifer*) and observed very large zones of inhibition (31–80 mm). In our work, we detected significantly smaller inhibition zones which nonetheless support the claim that CSEO has significant potential for the control of microscopic filamentous fungi. Badawy et al. [37] reported a significant antifungal effect of CSEO against *B. cinerea* at low concentrations, and they believe that CSEO could be suitable for controlling this plant pathogen. Our findings are in agreement with this, as the effect against *B. cinerea* observed by us was very strong.

Elansary et al. [29] determined minimal inhibitory concentrations of CSEO for *B. subtilis* and reported MIC values of less than 250 µg/mL. Their results for *S. aureus* and *P. aeruginosa* were 500 µg/mL and 2000 µg/mL, respectively. Since the authors do not distinguish between MIC 50 and MIC 90 as in our work, it is difficult to compare the given values, but for *B. subtilis* their MIC values were lower than MIC 50 and MIC 90 in our work, for *S. aureus* and *P. aeruginosa*, on the other hand, the MIC values are significantly higher than MIC 50 and MIC 90 in our work. Rguez et al. [38] determined the MIC values of CSEO for *S. aureus* (50 µg/mL) and *P. aeruginosa* (250 µg/mL). The findings of these authors are significantly lower for *S. aureus* than in our work but the MIC value of 250 µg/mL for *P. aeruginosa* is in the range of our MIC values of 50 and MIC 90. Taghreed [39] determined MIC values of 90 for *C. albicans* (0.42 µg/mL), *C. krusei* (64 µg/mL), and *C. glabrata* (64 µg/mL) after the application of CSEO, and these values are significantly lower than those determined in our work. The differences between the referenced and our results could have been caused by the different essential oil soils as well as the different microbial strains used for the analyses.

Ismail et al. [40] found that CSEO showed significant growth inhibition of all tested fungal species (*Fusarium culmorum*, *Fusarium oxysporum*, *Fusarium equisiti*, *Fusarium verticillioides*, *Fusarium nygamai*, *Botrytis cinerea*, *Microdochium nivale* var. *nivale,* and *Alternaria* sp.). Moreover, they determined CSEO to be effective against *B. cinerea* in a lower concentration. This finding is in agreement with our observations. Pansera et al. [41] observed the inhibitory effect of CSEO against conidial germination of several microscopic fungi, including *B. cinerea,* at very low concentrations, and concluded that this EO has the potential for use as an antifungal agent. The findings of the authors of this study support our findings.

Reyes-Jurado et al. [42] evaluated the effect of the vapor phase of many essential oils as potential antimicrobial agents and concluded that the results are encouraging and suggest possible applications in food preservation. Kloucek et al. [43] observed that filamentous microscopic fungi were more sensitive to vapor application than bacteria and yeasts. This statement is in agreement with our findings since higher efficiency was observed for filamentous microscopic fungi at lower EO concentrations. In a work by Kačániová et al. [44], a significantly higher efficacy of vapor phase application of EO compared to contact application was found. This is confirmed by our findings as the efficiency was higher at lower concentrations compared to contact application. In their work, Vimalaha et al. [45] evaluated the effect of vapor phase CSEO against viruses and bacteria, reporting promising results and suggesting that certain components contained in CSEO make its effect more pronounced in the vapor phase. These results are in accordance with our findings on the effectiveness of the CSEO vapor phase.

Jose et al. [46], who used microscopy and the crystal violet test, observed a significant eradication of *Klebsiella pneumoniae* biofilm after CSEO application. Despite the use of different species of biofilm-producing bacteria and other methods, these results are in agreement with our findings on the suitability of CSEO for the control of biofilm-producing bacteria. Rehman [47] investigated the antibiofilm effect of CSEO against *B. subtilis*, *P. multocida*, and *E. coli* and reported a very strong inhibitory effect determined using the crystal violet spectrophotometric assay. Despite methodological differences, our findings are in agreement with this observation. Pedroso et al. [48] reported that CSEO showed antibiofilm activity against all *Candida albicans* species tested which indicated that it could be an adjuvant in the treatment of *Candida albicans* infections associated with biofilm. Their results were obtained using a minimum inhibitory concentration test for biofilm and the MTT assay. Although they did not use MALDI TOF to evaluate the antibiofilm activity, as was the case in our study, they came to the same conclusion on the suitability of CSEO for combating biofilm. Jose et al. also confirmed the efficacy of CSEO against biofilms. Our findings are consistent with the previously published results on the efficacy of CSEO against biofilms. Silva [49] reported the antibiofilm effect of CSEO against biofilm produced by *Candia* species using an MTT assay. Despite the differences in the methods used, the author came to the same conclusion that CSEO has the potential to inhibit biofilm. Pereira et al. [16] reported that the use of MALDI TOF MS is sensitive enough to detect phenotypic changes in biofilm progression, and is also able to detect some distinct characteristics related to the surface on which the bacteria were grown. Kırmusaoğlu [50] states that the use of MALDI TOF MS is a very suitable method to study biofilm because extracellular polymeric substances (EPS) do not only contain polysaccharides, but also proteins such as extracellular enzymes. These expressed proteins localized in the EPS matrix can be detected and characterized by MALDI TOF MS.

Langsi et al. [51], evaluated the insecticidal activity of CSEO against insect pests of maize noting high efficacy. Our findings are in agreement with those of these authors. Pinto et al. [52] observed strong insecticidal effects of CSEO against *T. absoluta* in a dose- and exposure-duration-dependent manner. These findings are consistent with our observations, since in our experiment the effect decreased as a function of the concentration used. Almadii and Nenaah [24] reported that CSEO caused significant insecticidal bioactivity against *Culex quinquefasciatus*. Our findings on insecticidal activity are in agreement with the findings of other authors. Saad et al. [53] evaluated the efficacy of CSEO at different concentrations (50, 100, 200, 300, and 400 μg/mL) against *Tribolium castaneum* individuals, recording efficacies ranging from 42% to 20%. Compared to this author, the efficacy of the essential oil tested by us was higher. Ulukanli et al. [54] also reported good insecticidal efficacy of CSEO against *Ephestia kuehniella*. The authors’ findings support our findings that EO has the potential to be used as an insecticidal agent. Borotova et al. [55] stated that essential oils are a suitable alternative to synthetic insecticides.

Studies on the antiproliferative activity of CSEO are scarce. However, some reports show that it exerts a cytotoxic effect against NB4, HL-60, and EACC cell lines with LC_50_ values in concentrations ranging from 333.79 μg/mL to 372.43 μg/mL [34]. The results obtained in our study show that CSEO has antiproliferative activity towards human breast cancer MDA-MB-231, colon cancer HCT-116 cell line, choriocarcinoma JEG-3, and chronic myelogenous leukemia K562 cell line, and at the same time does not affect the viability of the human lung fibroblast cell line MRC-5. Moreover, the obtained data imply that K562 cells are the most sensitive to the treatment, suggesting that the essential oil tested in our study could be used for the treatment of chronic myelogenous leukemia.

## 4. Materials and Methods

### 4.1. Essential Oil

*Cupressus sempervirens* essential oil (CSEO) was purchased for research purposes from the Slovak company Hanus s.r.o. The essential oil (obtained from plant leaf) was stored at 4 °C without access to light during the whole duration of the experiments.

### 4.2. Microorganisms

Microorganisms purchased from the Czech Collection of Microorganisms (Brno, Czech Republic) were used for the analysis of antimicrobial activity. Analyses were performed on three gram-negative bacteria (*Pseudomonas aeruginosa* CCM 3955, *Yersinia enterocolitica* CCM 7204, *Salmonella enterica* subsp. *enterica* ser. Enteritidis CCM 4420), and 3 gram-positive bacteria (*Bacillus subtilis* CCM 1999, *Staphylococcus aureus* subsp. *aureus* CCM 2461, *Enterococcus faecalis* CCM 4224,). Analyses were also performed on 4 species of yeasts of the genus Candia (*Candida krusei* CCM 8271, *Candida albicans* CCM 8261, *Candida tropicalis* CCM 8223, *Candida glabrata* CCM 8270). A biofilm-producing strain of *S. enterica* isolated from a chicken sample during previous studies was used for the analysis of antibiofilm activity. Antifungal activity was evaluated on 3 fungal species (*Penicillium citrinum*, *Botrytis cinerea*, and *Aspergillus flavus*) that were isolated from grape samples. The isolated biofilm-producing bacterial strain and the tested fungal species were subjected to 16S rRNA sequencing and were also identified by MALDI-TOF MS Biotyper.

### 4.3. Chemical Characterization of CSEO by Gas Chromatography/Mass Spectrometry (GC/MS) and Gas Chromatography (GC-FID)

The volatile detection of *C. sempervirens* essential oil has been performed using GC and GC/MS analysis. For this purpose, used was Agilent Technologies (Palo Alto, Santa Clara, CA, USA) 6890 N gas chromatograph. The chromatograph was equipped with corresponding quadrupole mass spectrometer 5975 B (Agilent Technologies, Santa Clara, CA, USA), and operated with an interfaced HP Enhanced ChemStation software (Agilent Technologies). Separation of volatile compounds was performed by employing the HP-5MS capillary column (30 m × 0.25 mm × 0.25 µm). A sample of essential oil has been prepared by dilution with hexane (10% solution), and the injection volume was 1 µL. As carrier gas helium 5.0 was used, with the flow rate of 1mL/min. The temperature of the split/splitless injector, the MS source, and the MS quadruple was set at 280 °C, 230 °C, and 150 °C respectively. The mass scan range was 35–550 amu at 70 eV. The solvent delay time was 3.2 min for essential oil sample analysis, while in the case of n-alkanes (C7–C35), the solvent delay time was sat at 2.1 min to obtain the retention index for n-heptane which is identified at 2.6 min. The temperature conditions of the performed analysis were set as follows: from 50 °C to 90 °C (with a rate of increase of 3 °C/min), held for 4 min at 90 °C, from 90 °C to 130 °C (with a rate of increase of 4 °C/min), held for 1 min at 130 °C, from 130 °C to 290 °C (with a rate of increase of 5 °C/min). The total run time was 60 min, and the split ratio was 40.8:1. Volatile constituents were identified by the means of their retention indices (RI) comparison, as well as the reference spectra reported in the literature and the ones stored in the MS library (Wiley7Nist) [56,57]. Using GC-FID with the same HP-5MS capillary column performed was semi-quantification of the components taking into consideration amounts higher than 0.1%.

### 4.4. Antioxidant Activity

#### 4.4.1. DPPH Assay

The antioxidant activity of CSEO was determined using 2,2-diphenyl-1-picrylhydrazyl (DPPH, Sigma Aldrich, Germany). A stock solution of DPPH (0.025 g/L DPPH dissolved in methanol) was diluted by adding methanol to an absorbance of 0.7 at 515 nm. The analysis was carried out in 96-well microplates where 190 µL of diluted DPPH solution (absorbance 0.7) was injected into the well followed by the addition of 10 µL of CSEO. The prepared plate was incubated for 30 min at laboratory temperature without access to light on a shaker plate (MS 3 digital, IKA^®^, Deutschland, Germany) at 1000 rpm. The antioxidant activity of CSEO was expressed using the percentage of DPPH radical inhibition. (A0 − AA)/A0 × 100 was used for the calculation, where A0 was the absorbance of DPPH and AA was the absorbance of the sample. The relationship between the antioxidant activity and the reference substance Trolox (Sigma Aldrich, Schnelldorf, Germany) dissolved in methanol (Uvasol^®^ for spectroscopy, Merck, Darmstadt, Germany) was calculated over the concentration range 0–5 µg/mL which served as a standard. After constructing the calibration curve, the total antioxidant activity was expressed as the TEAC value. The results were presented as mean values ± standard deviation (SD) of three independent measurements.

#### 4.4.2. ABTS Assay

ABTS [2,20-azinobis(3-ethylbenzothiazoline-6-sulfonic acid) diammonium] radical cation was generated according to the already described procedure [58]. The prepared radical cation was diluted prior to the analysis up to an absorbance value of 0.7 at 744 nm. The 190 μL of this solution was mixed with 10 μL of EO (in a 96-well microtiter plate) for 30 min with continuous shaking at 1000 rpm at room temperature in the dark. A decrease in absorbance at 744 nm was registered and the results are presented as a percentage of ABTS inhibition using the same equation as in the previous section. All measurements were performed in triplicate. Methanol was used as blank, and Trolox as the standard reference substance. Results were expressed as % of inhibition as well as according to the calibration curve of Trolox (TEAC). The results were presented as mean values ± standard deviation (SD) of three independent measurements.

### 4.5. Antibacterial Activity

#### 4.5.1. Disc Diffusion Method

The disk diffusion method was used to determine the antimicrobial activity in the form of inhibition zones of CSEO. The inoculum of each microorganism was prepared 24 h in advance using Mueller Hinton broth (MHB, Oxoid, Basingstoke, UK) at 37 °C for bacteria and Sabouraud dextrose broth (SDB, Oxoid, Basingstoke, UK) at 25 °C for yeasts. The pre-cultured inoculum was adjusted to an optical density of 0.5 McFarland standard (1.5 × 10^8^ CFU/mL) by dilution with distilled water using a densitometer (BIOSAN, Latvian Republic). 100 µL of treated inoculum was applied to Petri dishes (PD) with Mueller Hinton agar (MHA, Oxoid, Basingstoke, UK) and spread thoroughly with an L-stick. Sterile blank discs (Oxoid, Basingstoke, UK) with a diameter of 6 mm were then placed on the PD. 10 µL of CSEO was applied to each disc. Samples were placed in thermostats according to the respective conditions for bacteria (37 °C) and yeast (25 °C) for 24h. Cefoxitin (Oxoid, Basingstoke, UK) was used as a positive control for gram-positive bacteria, gentamicin (Oxoid, Basingstoke, UK) for gram-negative bacteria, and one antifungal fluconazole (Oxoid, Basingstoke, UK) was used as a positive control for microscopic filamentous fungi. A solution of 0.1% dimethyl sulfoxide (Centralchem, Bratislava, Slovakia) served as a negative control. The radii of the inhibition zones (from disc edge to zone edge) formed by CSEO were measured at three locations and the standard deviation was then calculated. To evaluate the inhibition zones formed by the antibiotic control, the diameter of the inhibition zone (from zone edge to zone edge, including the 6 mm disc) was measured. 

To assess the antimicrobial activity of CSEO, the criteria for very strong activity were an inhibition zone of more than 10 mm, for moderate activity an inhibition zone of more than 5 mm, and for weak activity an inhibition zone of less than 5 mm. All measurements were performed in triplicate.

#### 4.5.2. Minimum Inhibitory Concentration (MIC)

The inoculum of each microorganism was prepared 24 h in advance using Mueller Hinton broth (MHB, Oxoid, Basingstoke, UK) at 37 °C for bacteria and Sabouraud dextrose broth (SDB, Oxoid, Basingstoke, UK) at 25 °C for yeast. The precultured inoculum was adjusted to an optical density of 0.5 McFarland standard by dilution with the appropriate broth using a densitometer (BIOSAN, Latvian Republic). The microbial inoculum was cultured for 24 h in Mueller Hinton broth (MHB, Oxoid, Basingstoke, UK) at 37 °C for bacteria and Sabouraud dextrose broth (SDB, Oxoid, Basingstoke, UK) at 25 °C for yeast. 100 μL of modified inoculum with an optical density of 0.5 McFarland standard (McF) was injected into a 96-well microtiter plate. A concentration gradient of CSEO was generated by serial dilution with a concentration range of 500 μL/mL to 0.244 μL/mL in the wells. MHB/SDB with EO was used as a negative control, and inoculum without addition was used as a positive control for maximum growth. At time 0h, the plates were measured with a Glomax spectrophotometer (Promega Inc., Madison, WI, USA) at 570 nm. Subsequently, the plates were placed in thermostats for 24h at the respective temperatures.

After 24 h incubation, absorbance was again measured using the spectrophotometer at 570 nm. The growth of microorganisms after 24 h was calculated. Subsequently, MIC 50 and MIC 90 values were calculated using logit analysis. The test was performed in triplicate.

The minimum inhibitory concentrations of CSEO against three species of microscopic filamentous fungi (*Penicillium citrinum*, *Botrytis cinerea*, and *Aspergillus flavus*) were evaluated by a different method because mycelial growth is difficult to observe by the agar microdilution method. Four concentrations (500, 250, 125, and 62.5 μL /mL) of CSEO were prepared by dilution in 0.1 % DMSO solution. The inoculum from the 24-hour culture was adjusted to 0.5 McF (1.5 × 10^8^ CFU/mL) by dilution with distilled water. 100 μL of inoculum was applied to the PD with SDA spread with an L-stick. Sterile blank discs were placed on the PDs and 10 μL of the appropriate concentration of CSEO was applied. The dishes were placed in a thermostat at 25 °C for 5 days. After cultivation, the inhibition zone radii were measured, and the mean inhibition zone and standard deviation were calculated for the respective concentration and the microscopic filamentous fungus. Antimicrobial activity was performed in triplicate.

### 4.6. In Situ Antimicrobial Activity

Seven species of bacteria of which 4 are gram-negative (*P. aeruginosa*, *Y. enterocolitica*, *S. enterica* subsp. *enterica* ser. Enteritidis, biofilm-forming *S. enterica*), 3 gram-positive bacteria (*B. subtilis*, *S. aureus* subsp. *aureus*, *E. faecalis*), 4 yeasts (*C. krusei*, *C. albicans*, *C. tropicalis*, *C. glabrata*) and 3 filamentous microscopic fungi (*P. citrinum*, *B. cinerea*, *A. flavus*) were used to analyze the antimicrobial effect of CSEO in situ in the vapor phase. A commercially available vegetable species (kohlrabi) was used as a model food. MHA and SDA were poured into a 60 mm diameter PD according to the species of microorganism (also into the lid). A slice of the model food with a thickness of about 0.5 cm was placed on the agar. The microbial inoculum (preparation described above) was applied to the slice using a bacteriological needle puncture. Dilutions of CSEO in ethyl acetate prepared concentrations of 62.5–500 μL/mL. The test concentrations were applied in a volume of 100 μL on sterile filter paper and placed in a PD cap. The PDs were hermetically sealed and placed in thermostats according to the respective culture conditions of the microorganisms used for 7 days. 

After incubation, the experiment was evaluated using stereological methods in ImageJ software. The bulk density (vv) of bacterial colonies was estimated and the grid points that contained colonies (P) and those (p) that were in the reference space (growth medium used) were counted. The bulk density of bacterial colonies was therefore calculated as follows: vv (%) = P/p. The antibacterial activity of EO was defined as the percentage of bacterial growth inhibition (BGI):

BGI = [(C − T)/C] × 100

where C and T are the bacterial growth (expressed as volume/volume) in the control and treatment groups, respectively. Negative results represent growth stimulation.

### 4.7. Antibiofilm Activity

MALDI-TOF MS Biotyper mass spectrometry was used to evaluate the effect of plant essential oil on the degradation of the *Salmonella enterica* biofilm protein profile. The experiment was conducted in 50 mL polypropylene centrifuge tubes. MHB culture medium in a volume of 20 mL was added to the tubes, and then the test surface species were placed in the form of strips about 1 cm thick and 5 cm long. Our analysis was performed on a stainless-steel surface and a plastic surface. For the experimental groups, the culture medium was enriched and supplemented with 0.1% (*w*/*v*) CSEO. A bacterial inoculum of 100 μL adjusted to an optical density of 0.5 McF was added to both groups. The samples were placed on an incubation shaker (GFL 3031, Germany) at 37 °C and 170 rpm.

Samples were analyzed by MALDI-TOF MS Biotyper mass spectrometry on days 3, 5, 7, 9, 12, and 14 of the experiment. Biofilm samples were collected with a sterile cotton swab from both surfaces and applied to a MALDI-TOF metal target plate. Planktonic cells obtained from the culture medium were also subjected to analysis. From the culture medium, 300 μL were collected and centrifuged at 12,000 rpm for 1 min. The supernatant was poured off and the pellet was washed 3 times with 30 μL of ultrapure water. Finally, the clean pellet was resuspended in 30 μL of ultrapure water and 1 μL was applied to a MALDI-TOF target plate. The samples were allowed to dry at room temperature.

After drying, the samples were overlaid with 1 μL of the a-cyano-4-hydroxycinnamic acid matrix (10 mg/mL). After crystallization of the matrix, the samples were analyzed using a MALDI-TOF MicroFlex (Bruker Daltonics, Billerica, MA, USA) with linear and positive mode settings with a range of *m*/*z* 200–2000. Using automated analysis, the same similarities were used to generate a standard global spectrum (MSP). Based on the Euclidean distance, 19 spectra were generated in MALDI Biotyper 3.0 and subsequently merged into a dendrogram [59].

### 4.8. Insecticidal Activity

Thirty individuals of *Oxycarenus lavaterae* were placed in the PD with vents. A circle of filter paper was placed in the lid of the PD on which the appropriate concentration (50, 25, 12.5, 6.25, and 3.125%) of CSEO was prepared by dilution in 0.1% polysorbate solution in a volume of 100 μL. The PDs were sealed around the perimeter using parafilm and left at room temperature for 24 h. A 0.1% polysorbate solution was used as a control. After 24 h, the number of dead and live individuals was evaluated, and insecticidal activity was calculated. The experiment was carried out in triplicate. The values for LC50 and LC90 were calculated using Finney’s Probit Analysis [60].

### 4.9. Determination of Cell Viability (MTT Assay)

The following reagents and chemicals were used: Dulbecco’s Modified Eagle medium (DMEM), 10% fetal bovine serum (FBS), 0.4% Trypan blue, 0.25%, trypsin-EDTA, dimethyl sulfoxide (DMSO), 3-(4,5-Dimethylthiazol-2-yl)-2,5-diphenyltetrazoliumbromide (MTT), phosphate-buffered saline (PBS), All the chemicals and reagents used in this study were of the highest commercially available purity. 

The human lung normal fibroblast cell line (MRC-5), breast cancer cell line (MDA-MB-231), colon cancer cell line (HCT-116), choriocarcinoma cell line (JEG-3), and chronic myelogenous leukemia cell line (K562) were obtained from American Tissue Culture Collection. These cells were propagated and maintained in DMEM and supplemented with 10% FBS and a combination of antibiotics (100 IU/mL penicillin and 100 µg/mL streptomycin). The cells were grown in a 75 cm^2^ culture flask and supplied with 15 mL DMEM at a confluence of 70% to 80%. The cells were seeded in a 96-well microplate (10,000 cells per well) and cultured in a humidified atmosphere with 5% CO_2_ at 37 °C. After 24 h of cell incubation, 100 μL of medium containing various doses of treatment (1 µg/mLto 200 µg/)mL was added to each well of the microplate, and the cells were incubated for 24 h and 72 h, after which the evaluation of cell viability was performed. Non-treated cells were used as control. The stock solution was prepared in the concentration of 10 mg/mL, while during the experiment used were concentrations of 1, 10, 20, 50, 100, and 200 µg/mL. The essential oil of *Cupressus semperiverens* was used in experiments. Above mentioned concentrations were obtained from the first stock solution by adding a certain volume of DMEM. All concentrations were used in triplicate for all the methods.

The viability of the cells was determined using an MTT assay [61]. Briefly, the cells were plated at a density of 10,000 cells/ mL (100 /µLwell) in 96-well plates with DMEM. After a period of incubation (24 h), at a temperature of 37 °C and 5% CO_2_, the 6 different concentrations of essential oil (from 1 to 200 µg/mL) dissolved in DMEM, were added to each well (100 µL per well). The untreated cells (cultured only in a medium) served as a control. After 24 and 72 h of incubation, the cell viability was determined with an MTT assay where 20 µL of MTT (concentration of 5 mg/mL) was added to each well. MTT is a yellow tetrazolium salt that is reduced to purple formazan in the presence of mitochondrial dehydrogenase. During this reaction, which started approximately after three hours, the formed crystals were dissolved in 20 µL of DMSO. The color formed in the reaction was measured on an ELISA reader at a wavelength of 550 nm. The percentage of viable cells was calculated as the ratio between the absorbance at each dose of the treatment and the absorbance of the non-treated control multiplied by 100 to get a percentage. We also calculated the half-maximal inhibitory concentration (IC_50_), defined as the concentration of tasted essential oil that inhibited cell growth by 50% when compared to the control. The IC_50_ values were calculated from the dose curves by the software CalcuSyn, Version 2.0.

### 4.10. Statistical Data Evaluation

One-way analysis of variance (ANOVA) was performed using Prism 8.0.1 (GraphPad Software, San Diego, CA, USA) followed by Tukey’s test at *p* < 0.05. SAS^®^ version 8 software was used to process the data. MIC values (concentration that caused 50% and 90% inhibition of bacterial growth) were determined by logit analysis. All data regarding the determination of cell viability were evaluated using IBM-SPSS 23 software for Windows (SPSS Inc., Chicago, IL, USA). The data were presented as a mean ± standard error (S.E.M). The statistical significance was determined using a Paired Sample-T test. The level of statistical significance was set at * *p* < 0.05.

## 5. Conclusions

The results of our study show that CSEO obtained from the commercial company Hanus s.r.o. produced in Slovakia shows good biological activity. The chemical composition evaluation revealed that CSEO was rich in α-pinene and δ-3-carene. DPPH and ABTS assay showed the better radical scavenging potential of this sample compared to the IC_50_ value Trolox. Higher antibacterial efficacy was determined using the agar diffusion method compared to the disc diffusion method. The antifungal activity of CSEO was weak to moderate depending on the concentration used. Also, the antibiofilm effect of this essential oil was demonstrated indicating its suitability as an alternative substance for combating biofilm-producing pathogens. The vapor phase effect was in most cases more pronounced at lower concentrations, but an inhibitory effect was observed for all the tested microorganisms at all tested concentrations, except for *S. enterica*. Based on our findings, we believe that CSEO could find application in storage extension and the protection of agricultural products in vapor application. The relatively strong insecticidal activity offers the possibility for the future use of CSEO in the control of agricultural insect pests. Results of cell viability testing showed that the viability of exposed cancer cells was significantly decreased compared to control cells in a time- and dose-dependent manner, and multifold lower when compared to non-cancer human lung fibroblast cells MRC-5. K562 cells were found to be the most sensitive to the treatment with CSEO, which suggests that the tested essential oil could be effectively used for the treatment of chronic myelogenous leukemia. Nonetheless, further tests are needed. 

## Figures and Tables

**Table 1:** Chemical composition of CSEO.

No	RT	RI (lit)	RI (calc.) ^a^	Compound ^b^	%
				Monoterpenes	
				*monoterpene hydrocarbons*	
1	7.22	926	925	Tricyclene	0.2
2	7.33	930	929	α-Thujene	0.9
3	7.69	939	940	α-Pinene	40.5
4	8.01	952	952	α-Fenchene	0.9
5	8.16	954	953	Camphene	0.5
6	8.28	967	957	Verbenene	tr ^c^
7	8.98	975	975	Sabinene	2.1
8	9.17	979	980	β-Pinene	1.7
9	9.60	990	990	β-Myrcene	3
10	10.45	1011	1012	δ-3-Carene	24.4
11	10.70	1017	1018	α-Terpinene	0.8
12	10.80	1024	1021	*p*-Cymene	Tr
13	11.02	1026	1026	*o*-Cymene	0.8
14	11.23	1029	1032	Limonene	4.3
15	11.29	1029	1033	β-Phellandrene	0.4
16	11.48	1050	1048	(*E*)-β-ocimene	Tr
17	12.43	1059	1060	γ-Terpinene	1.2
18	13.42	1088	1086	α-Terpinolene	8.6
19	13.83	1091	1090	*p*-Cymenene	Tr
				*Subtotal*	90.3
				*oxygenated monoterpenes*	
				*monoterpene ethers*	
20	11.35	1031	1035	1,8- Cineole	Tr
21	23.30	1244	1242	Carvacrol methyl ether	Tr
				*summ*	Tr
				*monoterpene alcohols*	
22	14.31	1096	1099	Linalool	0.5
23	16.48	1139	1140	trans-Pinocarveol	Tr
24	19.02	1177	1182	4-Terpineol	1.9
25	19.39	1182	1188	*p*-Cymen-8-ol	Tr
26	19.86	1188	1191	α-Terpineol	0.5
				*summ*	2.9
				*monoterpene ketones*	
27	16.89	1146	1145	Camphor	tr
28	17.48	1159	1157	Karahanaenone	0.3
29	18.33	1171	1171	Umbellulone	tr
				*summ*	0.3
				*monoterpene esters*	
30	24.30	1285	1285	Isobornyl acetate	0.4
				*summ*	0.4
				*subtotal*	3.6
				sesquiterpenes	
				*sesquiterpene hydrocarbons*	
31	28.07	1375	1373	α-Ylangene	tr
32	29.69	1411	1412	α-Cedrene	0.6
33	29.85	1419	1417	(*E*)-Caryophyllene	0.5
34	30.01	1420	1421	β-Cedrene	0.1
35	31.17	1454	1453	α-Humulene	0.2
36	31.86	1484	1471	α-Amorphene	0.2
37	32.06	1481	1476	Germacrene D	0.6
38	33.26	1513	1511	γ-Cadinene	0.4
				*subtotal*	2.6
				*oxygenated sesquiterpenes*	
				*sesquiterpene alcohols*	
39	35.89	1600	1608	Cedrol	2.4
				*subtotal*	2.4
				total	98.9

^a^ Values of retention indices on HP-5MS column; ^b^ Identified compounds; ^c^ tr-compounds identified in amounts less than 0.1%.

**Table 2:** In vitro antioxidant activity of CSEO.

	% of Inhibition	TEAC (mg/L)	Trolox (IC_50_) (mg/L)
DPPH^•^	76.32 ± 0.43	6.46 ± 0.18	4.39 ± 0.13
ABTS^•+^	91.53 ± 0.16	4.92 ± 0.06	2.96 ± 0.01

**Table 3:** Antimicrobial activity of CSEO with disc diffusion method.

Microorganism	Inhibition Zone	Activity of EO	Control
Gram-negative bacteria			
*Pseudomonas aeruginosa*	4.33 ± 1.15	*	25.00 ± 0.03
*Yersinia enterocolitica*	5.67 ± 0.58	**	24.00 ± 0.08
*Salmonella enterica* subsp. *enterica* ser. Enteritidis	6.67 ± 0.58	**	28.00 ± 0.06
*Salmonella enterica* biofilm	4.00 ± 1.00	*	25.00 ± 0.02
Gram-positive bacteria			
*Bacillus subtilis*	10.33 ± 1.53	***	26.00 ± 0.05
*Staphylococcus aureus* subsp. *aureus*	2.67 ± 0.58	*	24.00 ± 0.08
*Enterococcus faecalis*	1.00 ± 0.00	*	25.00 ± 0.08
Yeasts			
*Candida krusei*	3.67 ± 0.58	*	24.00 ± 0.09
*Candida albicans*	11.33 ± 1.15	***	26.00 ± 0.08
*Candida tropicalis*	6.33 ± 0.58	**	25.00 ± 0.02
*Candida glabrata*	8.33 ± 0.58	**	28.00 ± 0.04
Fungi			
* Aspergillus flavus *	6.33 ± 0.58	**	29.00 ± 1.00
* Botrytis cinerae *	6.67 ± 0.58	**	30.00 ± 1.00
* Penicillium citrinum *	7.67 ± 0.58	**	27.00 ± 1.50

* Weak activity (zone 1–5 mm); ** Moderate activity (zone 5–10 mm); *** Strong activity (over 10 mm); antibiotics used as a control: cefoxitin for G^−^ bacteria, gentamicin for G^+^ bacteria, fluconazole for microscopic filamentous fungi.

**Table 4:** Antimicrobial activity of CSEO.

Microorganism	MIC50 (µL/mL)	MIC90 (µL/mL)
Gram-negative bacteria		
*Pseudomonas aeruginosa*	202.11	401.67
*Yersinia enterocolitica*	93.80	99.91
*Salmonella enterica* subsp. *enterica* ser. Enteritidis	40.92	82.87
*Salmonella enterica* biofilm	187.31	199.21
Gram-positive bacteria		
*Bacillus subtilis*	374.02	397.64
*Staphylococcus aureus* subsp. *aureus*	202.11	401.67
*Enterococcus faecalis*	248.24	413.93
Yeasts		
* Candida albicans *	93.80	99.91
* Candida glabrata *	374.02	397.64
* Candida krusei *	71.30	131.49
* Candida tropicalis *	154.31	289.10

**Table 5:** Minimum inhibitory concentrations of CSEO against microscopic fungi.

Fungi	Concentration *(*µL/mL)	Inhibition Zone (mm)
*P. citrinum*	500	7.67 ± 1.53
	250	7.33 ± 0.58
	125	7.00 ± 1.00
	62.5	6.33 ± 1.53
*B. cinerea*	500	4.67 ± 0.58
	250	7.33 ± 2.31
	125	7.67 ± 0.58
	62.5	8.67 ± 1.15
*A. flavus*	500	7.33 ± 0.58
	250	5.33 ± 0.58
	125	4.67 ± 0.58
	62.5	2.67 ± 0.58

**Table 6:** In situ analysis of the antimicrobial activity of the vapor phase of CSEO in kohlrabi.

Bacteria		Bacterial Growth Inhibition (%)			
		The Concentration of CSEO in μL/mL			
		62.5	125	250	500
Gram-negative	*P. aeroginosa*	33.59 ± 2.01 ^d^	22.86 ± 1.62 ^c^	12.52 ± 1.06 ^b^	4.60 ± 1.16 ^a^
	*Y. enterocolitica*	44.74 ± 0.95 ^d^	33.53 ± 1.97 ^c^	23.34 ± 1.50 ^b^	15.00 ± 2.26 ^a^
	*S. enterica*	84.82 ± 3.00 ^d^	64.78 ± 2.57 ^c^	43.85 ± 1.84 ^b^	23.63 ± 1.95 ^a^
	*S. enterica biofilm*	7.05 ± 1.10 ^d^	−25.00 ± 2.41 ^c^	−44.04 ± 1.62 ^b^	−76.03 ± 2.78 ^a^
Gram-positive	*B. subtilis*	45.53 ± 2.24 ^c^	15.22 ± 1.36 ^b^	6.69 ± 1.11 ^a^	86.93 ± 2.00 ^d^
	*S. aureus*	35.74 ± 1.06 ^c^	14.89 ± 2.25 ^b^	43.59 ± 2.21 ^d^	7.19 ± 1.33 ^a^
	*E. faecalis*	64.22 ± 1.29 ^d^	13.81 ± 2.70 ^a^	22.44 ± 0.55 ^b^	33.71 ± 1.70 ^c^
Yeasts	*C. albicans*	44.33 ± 2.21 ^c^	76.15 ± 2.25 ^d^	36.30 ± 1.91 ^b^	15.63 ± 2.11 ^a^
	*C. glabrata*	34.03 ± 1.53 ^c^	14.29 ± 1.45 ^a^	43.77 ± 2.05 ^d^	24.03 ± 1.28 ^b^
	*C. krusei*	54.33 ± 2.15 ^c^	24.51 ± 1.38 ^a^	43.78 ± 1.95 ^b^	74.96 ± 1.69 ^d^
	*C. tropicalis*	16.78 ± 1.95 ^a^	34.93 ± 2.79 ^c^	25.74 ± 2.81 ^b^	56.63 ± 2.68 ^d^
Microscopic fungi	*A. flavus*	64.48 ± 2.81 ^a^	54.93 ± 1.54 ^d^	13.60 ± 1.78 ^c^	23.67 ± 2.01 ^b^
	*B. cinerea*	65.36 ± 2.01 ^d^	46.77 ± 2.72 ^c^	34.48 ± 2.06 ^b^	24.96 ± 2.71 ^a^
	*P. citrinum*	65.54 ± 2.31 ^c^	54.34 ± 2.07 ^d^	44.18 ± 1.47 ^b^	24.03 ± 1.54 ^a^

One-Way ANOVA, Individual letters ^(a–d)^ in the upper case indicate the statistical differences between the concentrations; *p* ≤ 0.05; the negative values indicate a probacterial activity of the essential oil against the growth of microbial strains.

**Table 7:** Insecticidal activity of CSEO.

Concentration (%)	Number of Living Individuals	Number of Dead Individuals	Insecticidal Activity (%)
100	0	30	100.00
50	6	24	80.00
25	14	16	53.33
12.5	19	11	36.66
6.25	27	3	10.00
3.125	30	0	0.00
Control group	30	0	0.00

## Data Availability

All data generated or analyzed during this study are included in this published article.
